# Clinical management of a Nigerian patient affected by sickle cell disease with rare blood group and persistent SARS‐CoV‐2 positivity

**DOI:** 10.1002/jha2.53

**Published:** 2020-07-04

**Authors:** Micol Quaresima, Virginia Quaresima, Matteo Maria Naldini, Daniela Maria Cirillo, Angela Ferrari, Angela Mazzi, Ester Maria Carla Tesini, Maria Cristina Leone, Francesco Merli

**Affiliations:** ^1^ Haematology Department Azienda USL‐IRCCS di Reggio Emilia Reggio Emilia Italy; ^2^ Emerging Bacterial Pathogens Unit Division of Immunology Transplantation and Infectious Diseases IRCCS San Raffaele Scientific Institute Milan Italy; ^3^ San Raffaele Telethon Institute for Gene Therapy (SR‐Tiget) IRCCS San Raffaele Scientific Institute Milan Italy; ^4^ Transfusion Medicine Unit Azienda USL‐IRCCS di Reggio Emilia Reggio Emilia Italy; ^5^ Unit of Gastroenterology and Digestive Endoscopy Azienda USL‐IRCCS di Reggio Emilia Reggio Emilia Italy; ^6^ Medicina II Cardiovascolare Department of Internal Medicine Azienda USL‐IRCCS di Reggio Emilia Reggio Emilia Italy

**Keywords:** blood groups, infection, sickle cell disease

## 1 CASE PRESENTATION

This article describes the case of a SARS‐CoV‐2 infection in an 18‐year old female Nigerian homozygous sickle cell disease (SCD) patient with the expression of a rare blood group phenotype. The patient is the first‐born daughter of two sickle cell trait (SCT) carriers who have settled in Italy from Nigeria.

On March 6, 2020, the patient self‐presented to the emergency department (ED) of the *Arcispedale Santa Maria Nuova* (ASMN) of Reggio Emilia for high grade fever (body temperature > 39°C) and headache persisting for the past 24 h.

Upon physical examination, the patient had a body temperature of 37°C and normal oxygen saturation (SpO_2_ = 98%) in ambient air. Chest CT scan highlighted superior right lobe parenchymal thickening with minimal bronchial aerograms and three confined small areas (largest diameter < 26 mm) of ground glass opacities. No pleural effusion was detected.

Considering that by March 2020, the Italian health system was struggling with the implementation of a harmonized national public health strategy for the containment of the COVID‐19 outbreak, testing for SARS‐CoV‐2 was not performed, and, given her mildly symptomatic status, the patient was discharged with Azithromycin 500 mg/day for 6 days and paracetamol for fever and pain relief. The patient was instructed about distancing measures from the rest of her family and home‐quarantine.

The patient was previously known at the ASMN Hematology department for her homozygous SCD status with a blood group (BG) B Rh positive, and a rare phenotype characterized by: ccDee (Rh System); kk (Kell System); Fya‐b‐ (Duffy system); Jka+b‐ (Kidd system); M+N‐S‐s‐ (MNS system); Cw‐ (Cw system); Le a‐b‐ (Lewis system); Lu a‐b+ (Lutheran system); Kp a‐b‐ (Kp system). Patients of African descent with rare blood types may face difficulties when in need of chronic transfusions, because of scarcely available fully compatible blood products since certain antigens are very rare in the Caucasian population.[1] If blood units are transfused within different ethnic backgrounds there is a higher risk of developing red blood cell (RBC) alloantibodies, which represents a severe complication for proper management of SCD patients.

The past medical history of our patient was notable for a SCD related episode of a vaso‐occlusive crisis (VOC) in 2014 and left elbow joint effusion in 2017. In 2018, the patient suffered from acute intrahepatic cholestasis which resolved spontaneously, and since then, also in consideration of her rare BG, the patient was started on hydroxyurea (HU) prophylaxis (10 mg/kg/day for 5 days a week and 15 mg/kg/day on weekends). Past history for Acute Chest Syndrome (ACS) was negative.

On March 17^th^, the patient presented again to the ED for chest pain and was admitted to the short stay observation unit (SSOU) for 24 h. Electrocardiogram (EKG) was unremarkable and cardiac troponin dosing was negative. Pain management required i.v. antalgic therapy. Naso‐ and oro‐pharyngeal swabs were obtained, and SARS‐CoV‐2 positivity was confirmed by real‐time reverse‐transcription–polymerase‐chain‐reaction (RT‐PCR).

Laboratory blood tests revealed mild anemia (hemoglobin (Hb) of 8.8 g/dL, hematocrit 27.5%, mean corpuscular volume (MCV) 97 fl), and a platelet count of (PLT) 1217 × 1000/μL. White blood cell (WBC) count of 4.76 × 1000/μL with an absolute lymphocyte count of 1990/μL. Renal function and liver enzymes were within normal values, although LDH was not tested. Inflammatory profile revealed low C‐reactive protein (0.27 mg/dL; normal range 0.00‐0.50 mg/dL) and elevated D‐dimer (1454 ng/mL; normal range 10‐500 ng/mL).

In the absence of cough, fever, and respiratory distress (SoPO_2_ = 98%), ACS was likely excluded also in consideration that radiographic evidence of pulmonary infiltrates was already evident much earlier (>48 h) than the onset of chest pain. HbS dosing was not performed because it is not among the routine emergency examinations. The patient was discharged with the advice to continue home self‐isolation and clinical follow up was maintained through frequent phone calls. During the first phase of COVID‐19 outbreak, mildly symptomatic patients were more preferably cared for at home, rather than admitted to overwhelmed hospitals.

The patient tested again positive for SARS‐CoV‐2 on two occasions (March 31 and April 18, 2020) and a follow‐up chest CT scan (April 18, 2020) confirmed the persistence of a superior right lobe parenchymal consolidation.

On April 20, 2020 (47 days from symptoms’ onset), due to the enduring SARS‐CoV‐2 positivity along with radiological abnormalities at chest CT scan, the patient was admitted to the internal medicine department. The pulmonary consolidation was interpreted as a consequence of SARS‐CoV‐2 infection and subcutaneous heparin at therapeutic dose (4000 UI/sQ b.i.d.) was initiated [2], along with IV ceftriaxone therapy (2 g). Urine legionella and pneumococcal antigen tests resulted negative. Normal plasma IL‐6 levels (0.0‐7.0 pg/mL), slightly increased lactate dehydrogenase (LDH) values (495 U/L; normal range 208‐378 U/L), and normal C‐reactive protein (0.19 mg/L) were reported. Oxygen therapy was not needed as the patient maintained SpO_2 _= 98% and pO_2_/FiO_2 _= 472 mm Hg at arterial blood gas (ABG) analysis in ambient air.

On day 3 of hospitalization, laboratory tests evidenced systemic hemolysis with a 1 g/dL decrease in hemoglobin in 48 h, and Hb‐S fraction of 75.5%. Tramadol (100 mg/i.v./b.i.d.) was substituted with continuous intravenous morphine (30 mg/i.v.) for pain control and a single unit of packed red blood cell (PRBC) (O Rh negative, ccdee) was transfused to dilute the HbS level. No transfusion related adverse events were recorded and HbS decreased to 62.5%.

On day 4, the patient experienced worsening uncontrolled pain crisis (Numeric Rating Scale[3] = 8) that required adjusting the intravenous morphine dose (40 mg/i.v./24 h) and HU therapy was increased to 20 mg/kg/day. On the same day, a first negative SARS‐CoV‐2 RT‐PCR was obtained, although the second confirmatory test (on April 26th) turned out positive.

On day 5, due to a plummeting platelet count (<50 000/μL) heparin was suspended. To exclude heparin‐induced thrombocytopenia (HIT), fondaparinux therapy was initiated and anti‐platelet factor 4 (PF4)/heparin antibodies tested negative. The patient's vital signs remained unremarkable. Hb level increased to 9 g/dL, and no alterations in hepato‐renal function and cardiac enzymes were evidenced, despite a persistently elevated D‐dimer (1077 ng/mL).

On day 8, the patient's continuous i.v. morphine dose was increased (50 mg/i.v., continuous infusion) for uncontrolled pain.

On May 1, 2020, a second blood transfusion was attempted (O Rh positive, ccDee) but had to be interrupted due to transfusion related adverse events (dizziness and general malaise).

On day 13 from hospitalization, the patient referred a reduction in perceived pain (NRS = 5) and morphine was reduced (30 mg/i.v.). Further investigations with an extensive infectious panel was undertaken for the pulmonary consolidation and CMV‐DNA, β‐d‐glucan, Parvo B19 IgM/IgG, QuantiFERON, all tested negative.

On May 6, 2020, after two consecutive negative SARS‐CoV‐2 swabs, the patient was discharged with a Hb of 9.5 g/dL, HbS of 61.9% (last measured on April 24, 2020), increased PLT count (792 000/μL), normal hepato‐renal function, and controlled pain intensity (NRS < 5); in consideration of the patient's poor compliance with self‐administration of subcutaneous heparin, she was started on 100 mg/day acetylsalicylic acid.

Of the patient's five near family members, initially only the 52‐years‐old father, carrier of SCT, was diagnosed positive for COVID‐19 with fever (38°C) and no cough or dyspnea. Chest X‐ray and CT scan revealed findings suggestive of COVID‐19‐induced pneumonia. Oral hydroxychloroquine (HCQ) home treatment was prescribed at a dosage of 400 mg/b.i.d. for the first day and 200 mg/b.i.d. for the following 4 days. Sixteen days later, despite being asymptomatic since treatment onset, the patient still tested positive for SARS‐CoV‐2 and achieved negativity only after 32 days from diagnosis. Eventually, also the 49‐years‐old mother, also carrier of SCT, tested positive for SARS‐CoV‐2 but remained asymptomatic.

## 2 DISCUSSION

In 2019, a new‐type coronavirus (SARS‐CoV‐2) was identified as the etiological cause of a severe acute respiratory syndrome. By the first trimester of 2020, this novel coronavirus disease (COVID‐19) had rapidly evolved into a global pandemic with 193 affected countries worldwide and about 4 618 821 confirmed cases (as of May 18, 2020) (https://www.who.int/docs/default-source/coronaviruse/situation-reports/20200518-covid-19-sitrep-119.pdf?sfvrsn=4bd9de25_4. Accessed May 19, 2020). Europe is amongst the most hardly hit regions and Italy suffered from one of the highest COVID‐19 death rates.

A key aspect of the COVID‐19 pandemic response has been to guarantee equal and fair access to (national) healthcare for all members of society. The International Organization for Migration (IOM) (https://www.iom.int/news/iom-informing-migrant-communities-italy-protection-covid-19. Accessed April 23, 2020) and World Health Organization (WHO) – Regional Office for Europe (http://www.euro.who.int/__data/assets/pdf_file/0008/434978/Interim-guidance-refugee-and-migrant-health-COVID-19.pdf?ua=1. Accessed April 23, 2020) called for a united response aiming at addressing the needs and rights of migrants living in all countries and settings during the COVID‐19 outbreak.

In 2019, Italy hosted 5 255 503 foreigners living within its borders, representing 8.7% of the total Italian resident population. Three regions in the north of Italy (Lombardia, Emilia Romagna, and Veneto) together account for the highest rate of foreigner citizens living in Italy (34.2%). These same three regions have suffered a tremendous burden from COVID‐19, with Lombardia accounting for 37.5% of the total COVID‐19 cases in Italy, Emilia Romagna for 12.1%, and Veneto for 8.5%.

The SARS‐CoV‐2 infection among immigrants living in Italy accounts for 6395 (5.1%) cases, with Nigerian nationality recorded for 2% of the total (https://www.epicentro.iss.it/coronavirus/sars-cov-2-sorveglianza-dati. Accessed May 18, 2020).

SCD is the most frequent hemoglobinopathy in Italy and 83% of foreign SCD patients, come from Africa and, often, are affected by severe homozygous forms of the disease [4].

Besides classical disease symptoms, SCD patients suffer also from aberrant endothelial interactions, systemic inflammation, and activation of the coagulation system, all relevant players in COVID‐19 pathophysiology [5]. SCD might thus be a potential risk factor for a more serious clinical manifestation of COVID‐19 as officially stated by the Italian Society of Thalassemia and Hemoglobinopathies (SITE) (http://www.site-italia.org/2020/covid-19_eng.php. Accessed April 23, 2020). Moreover, COVID‐19 might favor the occurrence of SCD related complications including acute chest syndrome (ACS) by exacerbating inflammatory responses in patients with a chronic multisystem disease background [6].

COVID‐19 infection has been already reported in SCD patients worldwide. In France, a 45‐years‐old SCD patient suffering from COVID‐19 has been successfully treated with Tocilizumab (TCZ), an anti‐human IL‐6 receptor monoclonal antibody [7]. Moreover, other SCD cases affected by SARS‐CoV‐2 in the United States [8, 9] and the Netherlands [10] population have been recently reported.

At the ASMN Hematology Department, 42 sickle cell patients are managed and all, except two, have African origins; among them 15 are affected by homozygous SCD (6 males, 9 females). Of all the followed patients, only the cases hereby described was diagnosed as SARS‐CoV‐2 positive and, in Italy, only two other COVID‐19 cases in SCD patients have been reported so far.

This case‐report aims to highlight the relevance of a prompt COVID‐19 diagnosis, especially in people affected by SCD to exclude and properly manage SCD related events. As in our SCD patient, ACS remained the main cause of morbidity, often triggered by infectious events. Prompt and early measures to prevent and treat ACS in the event of viral infections, such as COVID‐19, were employed. Due to the delayed SARS‐CoV‐2 testing, our patient did not receive HCQ treatment, although its efficacy is still under debate. Furthermore, the patient's rare blood group prohibited her from benefitting from exchange transfusions which could have more effectively reduced HbS fraction with the resolution of VOCs.

The clinical management of the 18‐year SCD patient was troubled by the persistence of the parenchymal pulmonary consolidation, which remains to be further investigated by follow‐up chest CT scan and bronchoalveolar lavage (BAL) or bronchial aspirate examination.

Noteworthy, both the SCD patient and the SCT carrier parent, despite the presence of pulmonary abnormalities at chest CT scan, never reported any respiratory symptoms.

Although there is limited data on the interactions between COVID‐19 and SCD, previous data from the H1N1 outbreak highlighted [8] increased risk of SCD‐related events such as ACS upon viral infection but whether SCD might influence the clinical manifestation of COVID‐19 is unknown. One may speculate that the chronic pulmonary hypoperfusion, as a consequence of the reiteration of VOCs in SCD patients, may slow down the COVID‐19 associated immune infiltrate recruitment and cytokine release. Therefore, we wonder if in addition to protecting factors such as sex and young age, the SCD background could have contributed to milder COVID‐19 manifestations. More data across different age categories in this particular population are needed to investigate whether the SCD background is linked to different manifestations of COVID‐19.

Lastly, a clear analysis will be needed to understand whether national health programs in Italy and elsewhere during the COVID‐19 pandemic properly responded to the needs of immigrants and patients suffering from chronic diseases.

## CONFLICT OF INTEREST

The authors declare that they have no conflict of interest.
